# Activity Profiles of Soccer Players During the 2010 World Cup

**DOI:** 10.2478/hukin-2013-0060

**Published:** 2013-10-08

**Authors:** Filipe Manuel Clemente, Micael Santos Couceiro, Fernando Manuel Lourenço Martins, Monika Ognyanova Ivanova, Rui Mendes

**Affiliations:** 1 RoboCorp, Coimbra College of Education – Polytechnic Institute of Coimbra, Portugal.; 2 Faculty of Sport Sciences and Physical Education – University of Coimbra, Portugal.; 3 RoboCorp, Engineering Institute of Coimbra – Polytechnic Institute of Coimbra, Portugal.; 4 Intituto de Telecomunicações, Covilhã, Portugal (IT).; 5 School of Computing, Edinburgh Napier University, Scotland.

**Keywords:** Soccer, match analysis, activity profile, player’s position

## Abstract

The main objective of this study was to analyse the distance covered and the activity profile that players presented at the FIFA World Cup in 2010. Complementarily, the distance covered by each team within the same competition was analysed. For the purposes of this study 443 players were analysed, of which 35 were goalkeepers, 84 were external defenders, 77 were central defenders, 182 were midfielders, and 65 were forwards. Afterwards, a thorough analysis was performed on 16 teams that reached the group stage, 8 teams that achieved the round of 16, 4 teams that reached the quarter-finals, and 4 teams that qualified for the semi-finals and finals. A comparison of the mean distance covered per minute among the playing positions showed statistically significant differences (F(4,438) = 559.283; p < 0.001; 2 = 0.836; Power = 1.00). A comparison of the activity time among tactical positions also resulted in statistically significant differences, specifically, low activity (F(4,183.371) = 1476.844; p < 0.001; 2 = 0.742; Power = 1.00), medium activity (F(4,183.370) = 1408.106; p < 0.001; 2 = 0.731; Power = 1.00), and high activity (F(4,182.861) = 1152.508; p < 0.001; 2 = 0.703; Power = 1.00). Comparing the mean distance covered by teams, differences that are not statistically significant were observed (F(3,9.651) = 4.337; p < 0.035; 2 = 0.206; Power = 0.541). In conclusion, the tactical positions of the players and their specific tasks influence the activity profile and physical demands during a match.

## Introduction

In sports, the performance profile of each player or team can be influenced by constraints related to both biological and environmental factors. From this it can be deduced that soccer performance depends on a countless number of factors (StØlen et al., 2006).

The kinematic analysis of soccer players during a match can provide useful information about their performance (Barros et al., 2007). A global index of physiological demands on players is represented by the total distance covered in a game (Reilly and Gilbourne, 2003).

The distance covered by players in a match, according to their positions, can be used to prescribe more specific training or to consider new ways to improve the efficiency of team training. With this perspective, several studies have analysed this particular variable (Di Salvo et al., 2007; Miyagi et al., 1999; Odetoyinbo et al., 2007; Rampinini et al., 2007; Reilly and Thomas, 1976).

In addition, some studies have analysed the distance covered by players taking into account their positions and then verified the observed differences (Braz et al., 2010; Dellal et al., 2011; Di Salvo et al., 2007; Mohr et al., 2003; Rampinini et al., 2007; Reilly & Thomas, 1976). In fact, the behaviour of each player is strongly influenced by the team’s specific strategy and tactical definition, as those determine the physical profile of the contemporary player in a professional match, especially in consideration of his individual position (Dellal et al., 2011). Moreover, some studies have presented unanimous differences between global positions (e.g., external defenders, central defenders, midfielders, and forwards) that show the importance of tactical position as a key factor in understanding the physical profile of players (Braz et al., 2010; Di Salvo et al., 2007).

Simultaneously with the analysis of the distance covered, the intensity of various activities during soccer games has been widely studied (Bangsbo et al., 1991; Braz et al., 2010; Castagna et al., 2003; Di Salvo et al., 2007; Reilly and Thomas, 1976). Some studies agree that it is better to measure physical performance during a soccer game (Impellizzeri et al., 2005; Mohr et al., 2003). In the analysis of the distance covered, the running intensity or activity profile of each player can depend directly on his position and tactical functions. Therefore, the distance covered at various speeds by elite soccer players depends on the contextual factors of the match (Lago et al., 2010).

The main objective of this study was to analyse the distance covered and the activity profile of soccer players in order to verify if performance variables are influenced by the tactical positions of players. Furthermore, the distance covered by each team has also been analysed to determine its possible influence on the level of performance exhibited by the competing teams.

## Material and Methods

### Sample

The data used in this study were obtained through the official website of FIFA World Cup 2010:http://www.fifa.com/worldcup/archive/southafrica2010/index.html). In terms of player-related data, the dependent variables of the distance covered, the distance covered while in possession of the ball, the distance covered while not in possession, the minutes played, and the activity for each player were obtained from this website. In terms of team-related data, the dependent variables of the distance covered, the distance covered while in possession, the distance covered while not in possession, and the number of matches played were obtained. The distance covered was measured in metres.

### General Procedures

#### Player Variables Analyzed

Position in the field is considered to be an independent variable. The players’ positions were divided into five groups: 1) goalkeeper; 2) external defender; 3) central defender; 4) midfielder (central and external); and 5) forward. For our study, the research sample consisted of 443 players, of whom 35 were goalkeepers, 84 were external defenders, 77 were central defenders, 182 were midfielders, and 65 were forwards.

This study considers an alternative perspective in the analysis of dependent variables. For the most part, studies of a similar design have analysed the distance based on the total sum of metres covered (Di Salvo et al., 2007; Rampinini et al., 2007). The analysis proposed in this paper simplifies the understanding of the dependent variable of the distance covered.

However, in order to allow for an accurate and fair comparison between the most common method and our own, the latter only considered players who played during the entire 90 minutes of each game. Thus, these methods reduce the opportunity to analyse the most probable number of players. To achieve this, a new procedure to interpret the dependent variables such as the distance covered or activity time was defined. Firstly, every player that played a minimum of 90 minutes in the 2010 World Cup was considered. Secondly, the dependent variables of distance covered, distance covered in possession, and distance covered not in possession were divided by the total amount of minutes played by each player. The result of this procedure shows the distance each player covered per minute.

Next, considering the aspect of the time spent at different levels of activity, the total amount of time spent in low-, medium-, and high-intensity activity was calculated on the basis of the data available on the official site. Nevertheless, the FIFA World Cup website does not show the standard levels that determine the type of intensity, thus reducing the possibility to compare these standards directly with other studies (Bangsbo, 1994; Barros, 2007; Reilly, 1993). Afterwards, each intensity level of activity was divided by the total time and the outcome was multiplied by 100. The final result presented the time percentage of each kind of activity.

#### Team Variables Analyzed

We considered the maximum stage reached by each team in the competition to be an independent variable, and distinguished four different stages: 1) group stage; 2) round of 16; 3) quarter-finals; and 4) semi-finals and finals. Our analysis included 16 teams that achieved the group stage, eight teams that reached the round of 16, four teams that reached the quarter-finals, and four teams that qualified for the semi-finals and finals.

In order to acquire the value of the mean distance covered in each match, the dependent variables of distance covered, distance covered in possession, and distance covered not in possession were divided by the number of matches played.

### Statistical Procedures

Due to the non-homogeneity of the sample assessed by the Levene’s test, the Central Limit Theorem was considered, which allowed us to adopt the assumption of normality (Akritas and Papadatos, 2004). Consequently, statistically significant differences between the dependent variables were established using the Welch Fw parametric test. This test was used because it usually shows better results for similar case studies (Pallant, 2011). In order to analyse the differences between the variables, the Games-Howell test was used as a post hoc test. Generally, this test is more effective than the other alternatives for case studies similar to ours. The estimation of the effect size, 2 (i.e., the proportion of the variance in the dependent variables that can be explained by the independent variables), was done according to Pallant (2011). Apart from the effect size, the power of the corresponding test was also presented. The analysis of the power of the test is a fundamental procedure to validate the conclusions reached in the inferential analysis (Pallant, 2011). This analysis was performed using IBM SPSS Statistics for a significance level of 5%.

## Results

### Results of the player’s analysis

The comparison of the mean distance covered per minute among the playing positions showed statistically significant differences (F(4,438) = 559.283; p < 0.001; 2 = 0.836; Power = 1.00). More specifically, the post hoc tests showed that midfielders covered the largest distance in comparison to goalkeepers (p < 0.001), central defenders (p < 0.001), external defenders (p < 0.001), and forwards (p < 0.001). The position that showed the second largest distance covered was external defenders in comparison to goalkeepers (p < 0.001) and central defenders (p < 0.001), but not to forwards (p = 0.999). The results also indicated statistically significant differences between forwards and central defenders (p < 0.001). In brief, excluding the goalkeeper position for tactical reasons, the central defender position shows the least distance covered.

The analysis of the mean distance covered per minute while in possession among the playing positions showed statistically significant differences (F(4,161.687) = 398.850; p < 0.001; = 0.623; Power = 1.00). More specifically, post hoc tests showed that the largest distance while in possession was covered by midfielders in comparison to goalkeepers (p < 0.001), central defenders (p < 0.001), external defenders (p < 0.001), and forwards (p < 0.001). The position that showed the second largest distance covered while in possession was the forward in comparison to goalkeepers (p < 0.001), central defenders (p < 0.001), and external defenders (p < 0.001), but not to midfielders (p = 0.988). Statistically significant differences were also observed between external defenders and central defenders (p < 0.001). Once again, excluding the goalkeeper position, the central defender position showed the least distance covered in possession.

The comparison of the mean distance covered per minute while not in possession among the playing positions showed statistically significant differences (F(4,161.341) = 428.872; p < 0.001; = 0.642; Power = 1.00). More specifically, post hoc tests showed that midfielders covered the largest distance in comparison to goalkeepers (p < 0.001), central defenders (p < 0.001), external defenders (p = 0.015), and forwards (p < 0.001). The position that showed the second largest distance covered while not in possession was the external defender in comparison to goalkeepers (p < 0.001), central defenders (p = 0.030), and forwards (p < 0.001). Statistically significant differences were also observed between forwards and central defenders (p = 0.019). Excluding the goalkeeper position, the forward position showed the least distance covered while not in possession.

A comparison of time percentage spent in low-intensity activity among the playing positions showed statistically significant differences (F(4,183.371) = 1476.844; p < 0.001; 2 = 0.742; Power = 1.00). More specifically, post hoc tests showed that goalkeepers spent more time in low-intensity activity in comparison to other positions (p < 0.001). The position that showed the second largest amount of time spent in low-intensity activity was the central defender in comparison to external defenders (p < 0.001), midfielders (p < 0.001), and forwards (p < 0.001). The position that showed the third largest amount of time spent in low-intensity activity was the forward position, which showed statistically significant differences when compared to midfielders (p < 0.001), but not when compared to external defenders (p = 0.488). The results presented statistically significant differences between external defenders and midfielders (p < 0.001). In brief, midfielders showed the least time spent in low-intensity activity.

A comparison of time percentage spent in medium-intensity activity among playing positions showed statistically significant differences (F(4,183.370) = 1408.106; p < 0.001; 2 = 0.731; Power = 1.00). More specifically, post hoc tests showed that midfielders spent more time in medium-intensity activity in comparison to other positions (p < 0.001). The position that showed the second largest amount of time spent in medium-intensity activity was the external defender in comparison to all other positions (p < 0.001), with the exception of the forward position (p = 0.120). The position that showed the third largest amount of time spent in medium-intensity activity was the forward, which revealed no difference in relation to the central defender (p = 0.173). In brief, excluding the goalkeepers for tactical reasons, central defenders showed the least amount of time spent in medium-intensity activity.

Comparison of a high-intensity activity profile among the playing positions showed statistically significant differences (F(4,182.861) = 1152.508; p < 0.001; = 0.703; Power = 1.00). More specifically, post hoc tests showed that midfielders spent more time in high-intensity activity in comparison to other positions (p < 0.001). The position that showed the second largest amount of time spent in high-intensity activity was the external defender compared to others (p < 0.001), except for the forward position (p = 0.884), which showed the third largest amount of time spent in high-intensity activity and revealed no difference in relation to the central defender (p = 0.001). Therefore, excluding the goalkeepers, central defenders showed the least time spent in high-intensity activity.

#### Results of the team’s analysis

A comparison of the mean distance covered among the teams showed statistically insignificant differences (F(3,9.651) = 4.337; p < 0.035; 2 = 0.206; Power = 0.541). More specifically, post hoc tests showed differences between teams that did not move beyond the group stage and teams that reached the semi-finals and/or finals (p = 0.007).

Comparing the mean distance covered in possession by the different teams did not show any statistically significant differences (F(3,28) = 2.178; p < 0.113). However, it is possible to observe a positive relationship between the distance covered in possession and the stage achieved in competition. An increasingly higher possession time can be observed as teams advance in competition.

Finally, comparing the mean distance covered by teams while not in possession did not show any statistically significant differences (F(3,28) = 0.535; p < 0.662). It is noteworthy that teams that achieved the quarter-finals showed less time spent without possession. The second group that demonstrated this tendency included the teams that reached the semi-finals and finals.

## Discussion

The physical profile of players in professional team sports has been well described, especially in relation to individual playing positions (Dellal et al., 2011). The main objective of this study was to analyse the variables that were influenced by tactical positions at the 2010 World Cup. Also, the distance covered by teams was analysed in order to determine the characteristics of the best teams. The distance covered by the players in each game varies according to the position played. It has been reported that the highest distances are covered by midfield players, while central defenders usually cover the least distance (Reilly et al., 2008).

In support of this fact, the work of Mohr et al. (2003) shows that midfield players and forwards cover a larger total distance than defenders.

This study confirms that midfielders cover the most distance, followed by external defenders. Generally, the greatest distance covered by players is achieved by midfielders because those players act as links between defence and offence (Bloomfield et al., 2007; Reilly & Thomas, 1976). Midfielders are therefore of essential importance to a team’s connectivity since the statistical analysis shows that they tend to cover the largest distance while the team is in possession.

Bangsbo (1994) reported that elite defenders and forwards cover approximately the same mean distance, which is significantly less than the distance covered by midfield players. This study shows that central defenders, excluding goalkeepers, cover considerably less distance than any other tactical position. However, by analysing the distance covered while the team is not in possession, it is possible to observe that forwards cover the least distance. Therefore, it can be concluded that forward is the position that covers the smallest distance in defensive manoeuvres.

To confirm this statement, it was necessary to use tactical metrics to observe the real participation and efficiency of forwards during defensive manoeuvres, since the distance covered did not provide an adequate understanding by itself.

Furthermore, the players’ activity profiles were analysed since high-intensity activity was suggested to be the best measure of physical performance during a soccer game (Mohr et al., 2003; Impellizzeri et al., 2005). Several studies have also demonstrated that soccer requires that participants repeatedly perform short-duration actions at maximal or submaximal intensity with brief recovery periods (Ferrari Bravo et al., 2008; Spencer et al., 2005). In fact, the majority of the distance was covered by sustained submaximal effort (Catterall et al., 1993).

Generally, elite soccer players cover the majority of the distance they cover during a match at a low intensity of activity (Rienzi et al., 2000). Indeed, the study by Rienzi et al. (2000) has shown the minimum activity profile percentage of midfielders to be 79.68%. Also, it is possible to confirm that, with the exception of goalkeepers, central defenders demonstrate the highest percentage of low-intensity activity time (85.87%), which is to say that they play most of the match at a low intensity. However, other studies show that, depending on their tactical positions, players cover different distances at different intensities (Di Salvo et al., 2009; Rampinini et al., 2007).

Defenders perform the largest amount of jogging, skipping, and shuffling movements and spend a significantly smaller amount of time sprinting and running than other players (Bloomfield et al., 2007). This observation is confirmed in the present study, which shows that central defenders spend less time in medium- and high-intensity activity. A similar situation was found in a study by Bangsbo (1994) in which defenders were observed to cover a smaller total distance with high-intensity running than other players. This is probably due to the tactical roles of defenders and their lower physical capacity. However, the lateral defenders also sprint and run. This could be related to the tactical roles of external defenders who are often required to perform sprints in both defensive and attacking phases (Di Salvo et al., 2010). Hence, it is possible to conclude that, immediately after the midfielder, the position that spends the most time in medium- and high-intensity activity is the external defender. However, midfielders and forwards also cover a larger distance in high-intensity running than defenders (Mohr et al., 2003). A greater sprinting distance is required not only of external defenders, but of wide midfielders and forwards as well (Di Salvo et al., 2009).

In the case of team analysis, the relevance of aerobic fitness for soccer players has also been confirmed by other studies which show a relationship between aerobic capacity and the ranking of teams (WislØff et al., 1998). However, for the purpose of the current study, all of the teams studied were ranked at a high level, as they all reached the World Cup.

It is also possible to notice that there is an increase in the distance covered while in possession in relation to the progression of a team in competition: that is, the more the team advances in competition, the longer the time that it is in possession of the ball.

It can be suggested that teams that achieved the highest stage in competition, also covered the longest distance while in possession. This could possibly be due to the quality and style of play, and it could also be related to the strategy implemented in each game. This strategy may depend on the stage of competition and the teams’ need to achieve their goals. Consequently, strategy attributes a fundamental weight to the influence of kinematic variables.

In brief, in a highly competitive playing environment such as the World Cup, the distance covered should not be the main factor in determining a team’s success. Other relevant factors such as the collective technical and tactical performance should also be taken into account. In addition to the kinematic variables, this study suggests new metrics for analysis of the teams’ collective behaviour in order to ensure a better understanding of the complex series of interrelations between numerous performance variables (Borrie et al., 2002).

In conclusion, it can be stated that novel methods complementing the kinematic analysis with tactical information will be an important tool for establishing new ways of training and improving the quality of the strategic approach to the game (Clemente et al., 2012).

## Conclusions

The purpose of this study was to analyse certain differences among playing positions and to quantify the demands placed on soccer players in each of the individual positions during the 2010 World Cup matches. Additionally, the distance covered by the teams was analysed. Statistically significant differences among tactical positions were found, concluding that each position has its specific demands. The variables of the strategic and specific missions of tactical disposition proved important for the understanding of two aspects – the demands placed on players during a game and how coaching intervention could be improved.

## Figures and Tables

**Figure 1:**
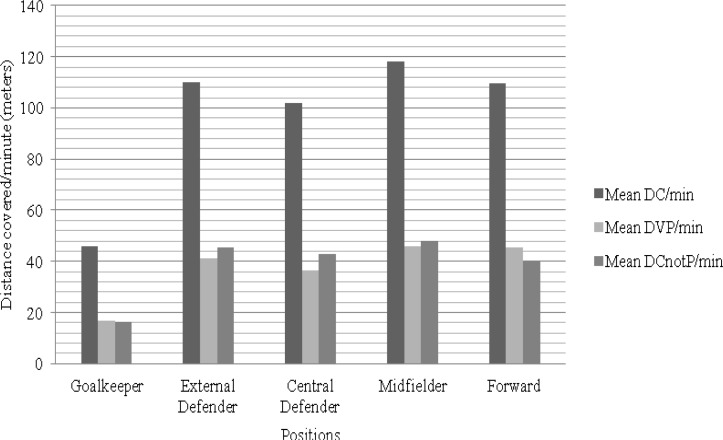
Graphical representation of the distance covered by players of different formation

**Figure 2:**
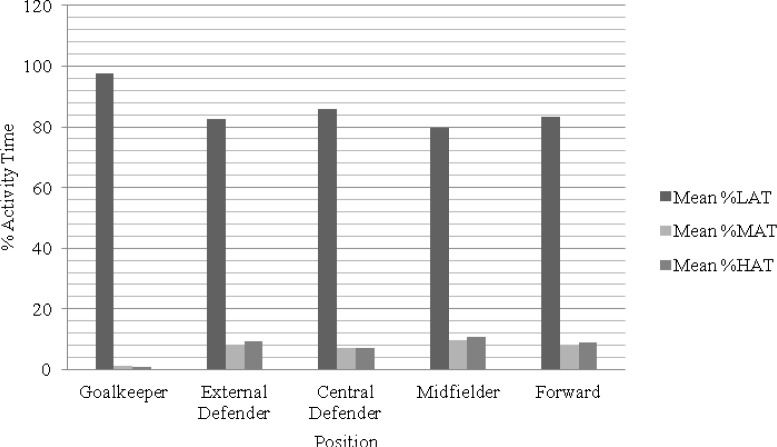
Graphical representation of the activity time of players of different formation

**Figure 3:**
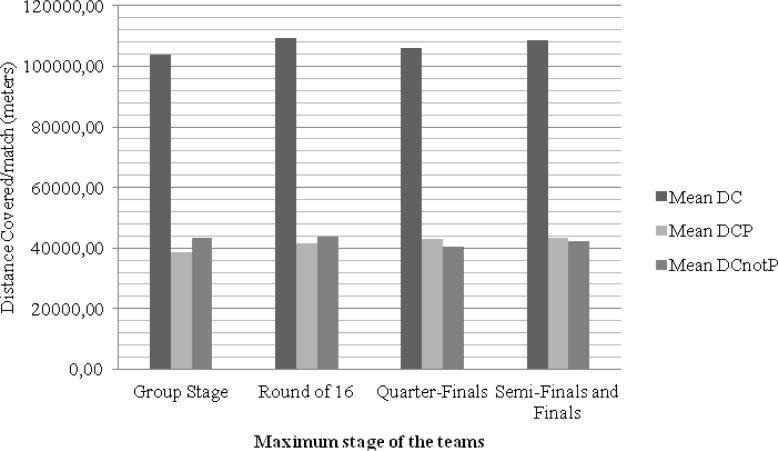
Graphical representation of the distance covered by teams reaching different stages of the 2010 World Cup

**Table 1:** Descriptive statistics of distance covered by players of different formation

	Mean			Standard Deviation			Minimum			Maximum		
Positions	DC/min	DVP/min	DCnotP/min	DC/min	DVP/min	DCnotP/min	DC/min	DVP/min	DCnotP/min	DC/min	DVP/min	DCnotP/min
Goalkeeper	45,69	16,74	16,4	8,999	3,526	3,704	30	10	10	67	26	27
External Defender	110,05	41,11	45,46	8,078	6,501	5,529	84	25	30	133	62	59
Central Defender	101,88	36,51	43	7,037	4,715	5,107	86	26	34	122	51	60
Midfielder	118,12	45,93	48,04	8,736	7,069	7,427	93	22	30	142	70	72
Forward	109,72	45,49	40,18	8,887	5,89	5,604	94	35	26	142	69	52
Global (Excluding GK)	109,94	42,26	44,17	8,185	6,044	5,917	89,25	27	30	134, 75	63	60,75

**Table 2:** Descriptive statistics of activity time of players of different formation

	Mean			Standard Deviation			Minimum			Maximum		
Positions	%LAT	%MAT	%HAT	%LAT	%MAT	%HAT	%LAT	%MAT	%HAT	%LAT	%MAT	%HAT
Goalkeeper	97,75	1,25	1	0,834	0,405	0,508	95	1	0	99	2	3
External Defender	82,73	8,16	9,12	2,721	1,249	1,641	74	5	5	89	11	15
Central Defender	85,87	7,22	6,92	2,333	1,091	1,37	78	5	4	91	11	12
Midfielder	79,68	9,61	10,71	3,295	1,59	1,977	70	5	5	89	13	17
Forward	83,49	7,66	8,86	2,896	1,24	1,816	73	6	6	88	11	16
Global (excluding GK)	82,94	8,16	8,90	2,81	1,29	1,70	73,75	5,25	5	89,25	11,5	15

**Table 3:** Descriptive statistics of distance covered by teams reaching different stages of the 2010 World Cup

	Mean			Standard Deviation			Minimum			Maximum		
Maximum stage of the teams	DC	DCP	DCnotP	DC	DCP	DCnotP	DC	DCP	DCnotP	DC	DCP	DCnotP
Group Stage	103997,71	38773,54	43429,58	4559,37	4469,59	4665,02	92840	31403	36443	112563	46740	52570
Round of 16	109495,94	41448,44	43710,31	5573,52	3591,43	4782,03	101778	34833	37548	118370	45908	50778
Quarter-Finals	106091,50	42960,00	40321,50	8035,32	1768,18	6097,03	98786	40488	35160	115402	44548	47544
Semi-Finals and Finals	108615,00	43226,43	42209,64	948,88	4724,85	4744,73	107406	38007	36917	109627	48971	48103
Global	107050,04	41602,10	42417,76	4779,27	3638,51	5072,20	100202,5	36182,75	36517	113990,5	46541,75	49748,75
